# Exosomes Derived From Dendritic Cells Infected With *Toxoplasma gondii* Show Antitumoral Activity in a Mouse Model of Colorectal Cancer

**DOI:** 10.3389/fonc.2022.899737

**Published:** 2022-05-04

**Authors:** Jinmiao Lu, Nana Wei, Shilan Zhu, Xiaoyu Chen, Haiyan Gong, Rongsheng Mi, Yan Huang, Zhaoguo Chen, Guoqing Li

**Affiliations:** ^1^ Guangdong Provincial Key Laboratory of Zoonosis Prevention and Control, College of Veterinary Medicine, South China Agricultural University, Guangzhou, China; ^2^ Key Laboratory of Animal Parasitology of Ministry of Agriculture, Laboratory of Quality and Safety Risk Assessment for Animal Products on Biohazards (Shanghai) of Ministry of Agriculture, Shanghai Veterinary Research Institute, Chinese Academy of Agricultural Sciences, Shanghai, China; ^3^ Key Laboratory of Adolescent Health Assessment and Exercise Intervention of Ministry of Education, East China Normal University, Shanghai, China

**Keywords:** *Toxoplasma gondii*, exosomes, myeloid derived suppressor cells (MDSCs), dendritic cells (DCs), colorectal cancer (CRC), immunosuppression

## Abstract

Pathogen-based cancer therapies have been widely studied. Parasites, such as *Toxoplasma gondii* have elicited great interest in cancer therapy. Considering safety in clinical applications, we tried to develop an exosome-based immunomodulator instead of a live parasite for tumor treatment. The exosomes, called DC-Me49-exo were isolated from culture supernatants of dendritic cells (DCs) infected with the Me49 strain of *T. gondii* and identified. We assessed the antitumoral effect of these exosomes in a mouse model of colorectal cancer (CRC). Results showed that the tumor growth was significantly inhibited after treatment with DC-Me49-exo. Proportion of polymorphonuclear granulocytic bone marrow-derived suppressor cells (G-MDSCs, CD11b^+^Ly6G^+^) and monocytic myeloid-derived suppressor cells (M-MDSCs, CD11b^+^Ly6C^+^) were decreased in the DC-Me49-exo group compared with the control groups *in vitro* and *in vivo*. The proportion of DCs (CD45^+^CD11c^+^) increased significantly in the DC-Me49-exo group. Levels of interleukin-6 (IL-6) and granulocyte-macrophage colony-stimulating factor (GM-CSF) significantly decreased after treatment with DC-Me49-exo. Furthermore, we found that DC-Me49-exo regulated the lever of MDSC mainly by inhibiting the signal transducer and activator of transcription (STAT3) signaling pathway. These results indicated that exosomes derived from DCs infected with *T. gondii* could be used as part of a novel cancer therapeutic strategy by reducing the proportion of MDSCs.

## Introduction

Many strategies have been used in colorectal-cancer (CRC) therapy. In recent years, cancer therapies based on pathogens (viruses, bacteria, and parasites) have elicited great interest. In 2015, the first oncolytic virus was approved for melanoma therapy by the U.S. Food and Drug Administration (FDA) (1). Bacteria-based cancer therapy has been approved in clinical trials by both the FDA and the National Medical Products Administration (NMPA). Other team and other researchers have found that infection by parasites such as *Toxoplasma gondii* and *Plasmodium* can inhibit tumor growth *in vivo*. However, although parasitic infection can change the balance of tumor tolerance, live parasites in cancer treatment increase the risk of infection.

Exosomes are extracellular vesicles with lipid bilayer molecules. They can carry proteins, biologically active lipids, and RNA from donor cells to recipient cells, thereby establishing intercellular communication and changing the functions of the recipient cells (2). Exosomes from pathogen-infected host cells have an anti-infective effect. These exosomes carry an “infection” message to immune cells, triggering them to activate the immune response (3). Dendritic cell (DC)-derived exosomes display abundant major histocompatibility complex (MHC) class I/II molecules and T cell co-stimulatory molecules, meaning that such exosomes have potential antitumoral activity (4). Researchers have discovered that pathogen-infected macrophages carry pathogen-associated molecular patterns (PAMPs), which can activate the host’s immune response mechanism (5). Exosomes secreted by immature DCs (DC-exo) produce an antitumoral immune response only when co-injected with mature DCs or chemical adjuvants (6). DC-exo can be used only in combination with chemotherapeutic drugs or specific immunotherapies to achieve better effect in clinical trials (7). Previous research has also showed that intra-tumoral injection of exosomes derived from the plasma of *Plasmodium*-infected mice significantly reduces the tumor growth in Lewis lung cancer (LLC) (8).

Myeloid-derived suppressor cells (MDSCs), which are immature myeloid cells derived from bone marrow (BM), are one of the most important types of immunosuppressive cells, and can inhibit immune cell responses (9–11). Removing MDSCs from the tumor microenvironment (TME) in patients can improve the host immune system’s ability to attack tumors and improve the effect of immunotherapy. Research has confirmed that *Plasmodium* infection inhibits the expansion and activation of MDSCs in a murine LLC model (12). A live, non-replicating, non-toxic *T. gondii* uracil-deficient vaccine strain (cps) reverses tumor-induced immunosuppression and promotes the M1 macrophage phenotype by activating immune cell such as DCs to suppress the role of MDSCs, thereby causing the inhibition of tumors (13). Therefore, we attempt to introduce the mechanism that activates host cell immunity by *T. gondii* to stimulate antitumoral immunity, as well as to investigate the potential of this strategy in tumor immunotherapy.

In our previous study, the DCs-derived exosomes “edited” by *T. gondii* had good cell compatibility and could interfere with immunosuppression caused by tumors. In the current study, we hypothesized that exosomes derived from DCs infected with Me49 strain of *T. gondii* could inhibit the level of MDSCs in a mouse model of CRC to achieve tumor suppressive effects. To develop an exosome-based immunomodulator instead of live parasites for tumor therapy, we evaluated antitumoral activity of exosomes isolated from DCs infected with the Me49 strain of *T. gondii* in a mouse model of CRC.

## Materials and Methods

### Ethical Approvals

All animal experiments were approved by the Institutional Animal Care and Use Committee of the Shanghai Veterinary Research Institute, Chinese Academy of Agricultural Sciences (CAAS), Shanghai, China (IACUC approve number SHVRI–SZ–20200421-01).

### Sources of Cells, Parasites and Mouse

We purchased cell line of murine colorectal carcinoma (CT26 labeled with luciferase) from the Shanghai Institutes for Biological Sciences, Chinese Academy of Sciences (Shanghai, China). The cell culture medium used in this experiment was Roswell Park Memorial Institute (RPMI) 1640 supplemented with 1% penicillin-streptomycin solution and 10% fetal bovine serum (FBS), all purchased from Gibco (Thermo Fisher Scientific, Waltham, MA, USA) in a humidified atmosphere of 5% CO_2_ at 37°C.

The Me49 strain of *T. gondii* was preserved at the Key Laboratory of Animal Parasitology of Ministry of Agriculture (China), Laboratory of Quality and Safety Risk Assessment for Animal Products on Biohazards of Ministry of Agriculture, China.

We obtained 6-week-old female BALB/c mice from Shanghai SPF Biotechnology Co., Ltd (Beijing, China) and kept them at the SPF Experimental Animal Center of Shanghai Veterinary Research Institute, CAAS. Animals were housed in cages at 21 ± 1°C and 50–60% humidity, on 12 h-light-dark cycles, with enrichment items located in ventilated racks.

### Animal Experimental Model

We randomly divided the mice into two groups (n = 5 per group). There were no significant differences in body weight between groups. Mice were immunized with inactivated *T. gondii* Me49 strain. Then mice were subcutaneously injected in the axillary area with 5×10^6^ CT26 cell suspension.

### *T. gondii* Treatment of Mice With Colorectal Cancer

After immunizing mouse with *T. gondii* Me49 strain, mice were injected with 5×10^6^ CT26 cells. Seven days later, 100 tachyzoites of *T. gondii* Me49 strain were infected in CT26+Me49 group. The control group (CT26) was injected with PBS.

### qPCR Detection of *T. gondii* in Different Tissues

We performed quantitative polymerase chain reaction (qPCR) amplification of the 529-bp target gene in *T. gondii* as described below. DNA was extracted from different tissues of mice with CRC using a DNA Mini Kit (Qiagen, Hilden, Germany). We performed qPCR on QuantStudio5 PCR system (Applied Biosystems, Foster City, CA, USA). *T. gondii* from different tissues was detected by qPCR assay using the primers (forward primer: 5’-GCTCGCCTGTGCTTGGAG-3’, reverse primer: 5’-ATCTTCTCCCTCTC CGACTCTC-3’) and probe (probe sequence: 5’-TCGCTTCCCAACCACGCCACCC-3’). Briefly, the 20 μL reaction mixture contained 10 μL premix, 0.8 μL forward primer, 0.8 μL reverse primer, 0.2 μL probe, 0.2 μL Rox Reference Dye II (Tokyo, Shiga, Japan), 6 μL ddH2O, and 2 μL template of DNA. The major steps of qPCR included denaturation at 95°C for 10 min, followed by 40 cycles of denaturation at 95°C for 15s, annealing at 60°C for 1min, and extension at 72°C for 45s. We prepared a standard curve and took measurements in triplicate for each sample.

### Tumor Volume Measurement

Tumor volume = length × width^2^/2, where length represents the largest tumor diameter and width represents the perpendicular tumor diameter. Mice were categorized as dead for ethical reasons when the tumor volume exceeded 1500 mm^3^.

### Flow Cytometric Analysis

Mice with CRC treated with *T. gondii* or exosomes were sacrificed before the flow cytometry (FCM) analysis. Then, we separated the blood, spleen, and tumor tissues. T lymphocyte infiltration and the proportion of MDSCs were detected by FCM. After washing the tumor samples in RPMI 1640, they were cut into 1 mm^3^ tissue pieces and digested with RPMI 1640, containing Dispase^®^ II (1.5 U/mL, Sigma, USA), Collagenase D (1 mg/mL, No.11088858001; Roche, Basel, Switerland) and 0.2% DNase I (Roche, USA) at 37°C for 30 min according to the previous study (14) with some modifications. The digested material was passed through a mesh (70 μM) to remove clumps and the filtrate was washed twice and then centrifuged at 400 × *g* for 8 min at room temperature (RT) (14).

We used a lysed solution to lyse blood, after which we washed it with PBS and then centrifuged at 700 g for 5 min. To prepare the single-cell spleen suspension, the whole spleen was placed in a cell strainer and crushed. We next passed the cells through the 70 μM mesh to remove clumps and washed the filtrate twice, then centrifuged it at 400 g for 8 min at RT. Cell surfaces were stained in accordance with established methods (15). A total of 1×10^6^ cells were incubated for 30 min at 4°C with different combinations of the following antibodies: (FITC)-CD3, (BV421)-CD4, (PE)-CD8, (APC)-CD45, (FITC)-CD11b, (PE)-Ly6G, (APC-A)-Ly6C, (FITC)-CD45, (APC)-CD11b, (PE)-Ly6G, (APC-700)-Ly6C, (PC5.5)-CD11c, and (PB450)-F4/80. All the antibodies were purchased from Becton, Dickinson (San Jose, CA, USA). Following two washes with 1 mL staining buffer, the cells were resuspended in 200 μL staining buffer for analysis on a CytoFLEX flow cytometry (Beckman Coulter Life Sciences, Brea, CA, USA).

### Isolation of DC-Derived Exosomes

We obtained bone marrow-derived dendritic cells (BMDCs) from BM suspensions prepared from mouse femurs as described previously (16). Exosomes were purified from the supernatants of DCs infected with *T. gondii* by differential centrifugation as previously described (17). Briefly, we harvested supernatants of DCs infected with *T. gondii* from the DC-Me49-exo group, and those of control DCs from the DC-exo group. The different supernatants were centrifuged at 500 g for 10 min to remove cell debris and other small particles, and the supernatants was collected. Then, we centrifuged the supernatant at 16,500 *g* for 20 min, followed by filtration through a 0.22-μm filter (Millipore Sigma). Finally, supernatant solutions were ultracentrifuged at 120,000 *g* for 90 min, and the exosome pellet was resuspended with an appropriate amount of PBS. We measured the protein content of exosomes using a BCA protein assay kit (Thermo Fisher, USA). Exosomes were stored at −80°C for future use or directly used in co-culture experiments.

### Characterization of Exosomes

We observed the size, morphology, and distribution of exosomes by transmission electron microscope (TEM). Diluted exosomes were fixed for 15 min in 2.5% formaldehyde/glutaraldehyde (Solarbio, Beijing, China), and 0.1 M sodium cacodylate buffer. The samples were then placed on a 300-mesh carbon coated mesh for air drying. We stained the samples for 4 min with a negatively filtered microporous aqueous solution of uranyl acetate, and then washed them twice with 50% methanol/water. After air drying, samples were observed under TEM with accelerating voltage of 80 kV and spot size of 2. We diluted purified exosomes in PBS (10000×) and subsequently used them for size measurement and analysis on a ZetaView nanoparticle-tracking analyzer (Particle Metrix, Inningam Ammersee, Germany) to determine concentration (particles/mL) and particle size (nm). Exosomes were mixed with loading buffer and heated at 100°C for 10 min. We then loaded exosome samples on 10% SDS-PAGE gel, transferred the samples to PVDF (Billerica, MA, USA), and incubated them overnight in blocking buffer (1× PBST, 5% milk). After five washes with washing buffer (PBST), membranes were incubated for 1 h with monoclonal antibodies of CD63, CD9, TSG101 (Cambridge, MA, USA) in a buffer containing PBST, and 1% milk. After washing them with washing buffer, we incubated membranes for 1 h with secondary antibodies and detected signals on a ChemiDoc Touch Imaging (Bio-Rad, USA).

### Co-Culture Assays *In Vitro*

Mouse BM–derived MDSCs were obtained as previously described (18, 19). In brief, we flushed BM cells from the femurs and tibias of approximately 5-week-old BALB/c mice. Red blood cells (RBCs) were lysed with lysis buffer (Thermo Fisher). The BM cells were then cultured in RPMI-1640 supplemented with 10% FBS, 1% penicillin-streptomycin solution and stimulated with 40 ng/mL interleukin-6 (IL-6) and granulocyte-macrophage colony stimulating factor (GM-CSF) (Rocky Hill, NJ, USA) at 37°C for 4 d in a 5% CO_2_-humidified atmosphere. Then, we detected unique M-MDSCs and G-MDSCs using (FITC)-CD45, (APC)-CD11b, (PE)-Ly6G, and (APC-700)-Ly6C. Uniquely DCs and macrophages were detected by (FITC)-CD45, (PC5.5)-CD11c, and (APC)-CD11b, (PB450)-F4/80. All the antibodies were purchased from Becton, Dickinson (San Jose, CA, USA). After induction and maturation, MDSCs were harvested. Then, MDSCs incubated with exosomes of DC-exo, DC-Me49-exo, and PBS, respectively. After 24 h, MDSC was collected for flow cytometry and Western blot. All cells were lysed using RIPA buffer (Beyotime Institute of Biotechnology, China) with 1 nM PMSF, and total protein concentration was determined using a BCA protein quantification kit (Thermo Fisher). Next, we loaded 5 µg total proteins per lane and resolved them on 0.6–0.8% gels by SDS-PAGE. Proteins were transferred onto PVDF membranes and blocked for 1 h with 5% non-fat milk at RT, after which the membranes were washed three times with PBST. Membranes were incubated for 1 h with monoclonal antibodies of P-JAK2, JAK2, P-STAT3, STAT3 and tubulin in a buffer containing PBST and 1% milk. All primary antibodies were purchased from CST. After washing them with PBST, we incubated membranes for 1 h with goat anti-mouse IgG-HRP (Santa Cruz, CA, USA) and detected signals on a ChemiDoc Touch Imaging (Bio-Rad, USA).

### Indirect Immunofluorescence

Exosomes were labeled by ExoSparker Exosome Membrane Labeling Kit-Green (Kumamoto, Japan), MDSCs were seeded into a 6-well plate at a density of 3×10^5^ cells per well and 5 μg/mL exosomes from each of the different groups were added to the MDSC culture medium. Control wells contained cells but no exosomes. After incubation for 24 h, we washed cells three times with PBS and stained them with DAPI (Beyotime Biotechnology, China). All cells were sealed and imaged using Zeiss LSM 880 confocal microscopy.

### Treatment of Colorectal Cancer-Bearing Mice With Exosomes

BALB/c female mice (6 weeks old) were weighed, randomly divided into three groups (n = 10 each group), and injected subcutaneously with 5 × 10^6^ CT26 cells. When the tumor was visible, treatment was carried out according to the following design: an intra-tumoral injection with 10 μg DC-exo (DC-exo group), 10 μg DC-Me49-exo (DC-Me49-exo group), and 10 μL PBS (PBS group). Two injections were performed on day 1 and 3 after tumor visualization (8). At day 19 post inoculation, the tumor progression was monitored by using an IVIS Spectrum imaging system (IVIS Spectrum, USA). Mice were sacrificed, and blood, spleens and tumors were collected for flow-cytometry analysis. Cytokines in serum were determined by using IL-6 and GM-CSF ELISA kits (Neobioscience Technology Company).

### Statistical Analysis

All data were analyzed using GraphPad Prism software (Version 8.0.2) and presented as mean ± standard deviation. The significant differences between experiment group and control group were analyzed using Student’s *t*-test or one-way ANOVA with Dunnett’s multiple comparison.

## Results

### *T. gondii* Infection Reduced Mortality and MDSC Levels in Tumor-Bearing Mice

**Figure 1:**
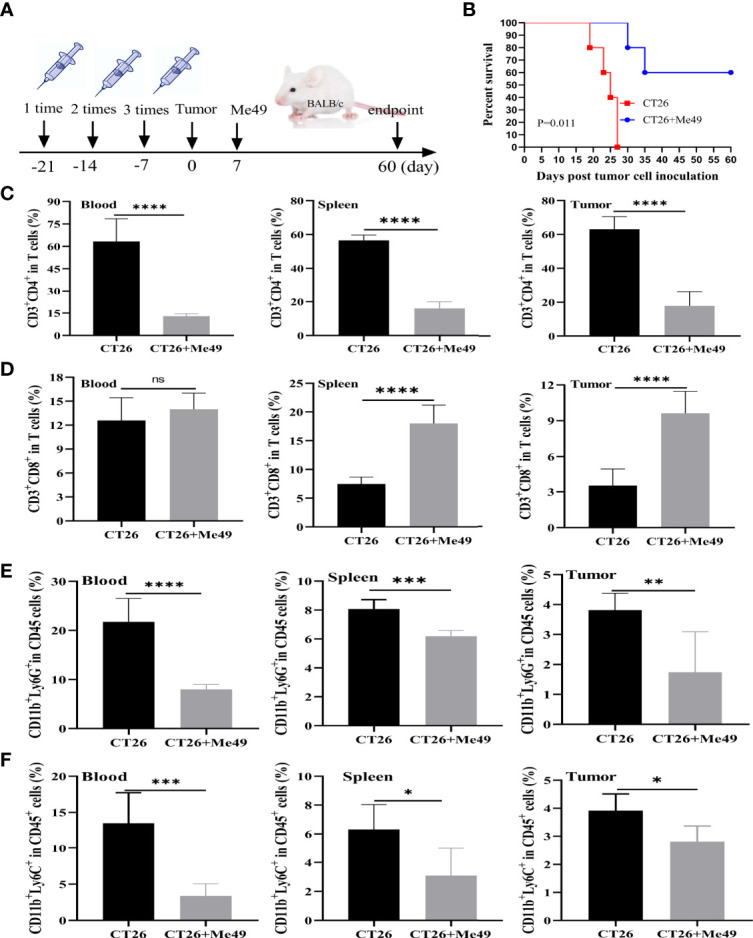
*T. gondii* induced a protective immune response against tumor growth of CRC. **(A)** Mice were immunized with inactivated Me49 strain of *T. gondii*, and then vaccinated with CT26 cells. After 7 d, mice were reinfected with the Me49 strain of *T. gondii*. The control group was not infected after tumor inoculation (n = 5). **(B)** Survival rate of CT26–bearing mice after *T. gondii* infection. FCM analysis of CD3^+^ CD4^+^ T cells (anti-CD3-FITC, anti-CD4-PB450) **(C)**, CD3^+^ CD8^+^ T cells (anti-CD3-FITC, anti-CD8-PE) **(D)**, G-MDSCs (anti-CD11b-FITC, anti-Ly6G-PE) **(E)** and M-MDSCs (anti-CD11b-FITC, anti-Ly6C-APC-A) **(F)** in peripheral blood, spleen and tumors from different groups. ns, (*p* ≥ 0.05), *p < 0.05, **p < 0.01, ***p < 0.001, ****p < 0.0001.

In order to determine the antitumoral activity of *T. gondii*, we established the mouse models of CRC. After being vaccinated three times with the heat-killed *T. gondii*, each mouse was inoculated with 5×10^6^ CT26 cells. Seven days later, the mouse was infected with the Me49 strain of *T. gondii* in the CT26 + Me49 group, and the CT26 group was injected with PBS (**Figure 1A**). Mice in the CT26 + Me49 group had a 60% survival rate at day 35 versus 0% for the untreated group (**Figure 1B**).

CD3^+^CD4^+^ and CD3^+^CD8^+^ T cells were evaluated using CytoFLEX. We used an FACS strategy (**Supplementary Figure 1A**) to isolate these cells from different tissues of tumor-bearing mice. In peripheral blood, spleen and tumor tissues of the CT26 + Me49 group, as compared with the control group, the mean frequency of CD4^+^ T cells was significantly reduced (*p* < 0.0001) (**Figure 1C**). In peripheral blood, no differences were found in the mean frequency of CD3^+^CD8^+^T cells between the CT26 group and the CT26 + Me49 group (**Figure 1D**). Compared with the CT26 group, the mean frequency of CD3^+^CD8^+^ T cells in both spleens and tumors was significantly increased in the CT26 + Me49 group (*p* < 0.0001) (**Figure 1D**). These data indicated that *T. gondii* infection could significantly increase the CD3^+^CD8^+^T cell infiltration into tumor tissue.

MDSCs demonstrate immune evasion, and promote tumor progression by inhibiting the proliferation and functions of T cells (20). In order to investigate whether infection with the Me49 strain of *T. gondii* could affect the level of MDSC, we prepared single-cell suspensions from the peripheral blood, spleen and tumor tissues, stained them with MDSCs-specific markers, and analyzed the cells using CytoFLEX. After CD45^+^CD11b^+^ gating (**Supplementary Figure 1B**), G-MDSCs and M-MDSCs were analyzed (n = 5/group). Compared with CT26 group, the G-MDSCs from peripheral blood (*p <*0.0001), spleens (*p* < 0.001), and tumors (*p* < 0.01) were significantly decreased in the CT26 + Me49 group (**Figure 1E**). Compared with CT26 group, percentage of M-MDSCs in peripheral blood (*p* < 0.001), spleens (*p* < 0.05) and tumors (*p* < 0.05) were also significantly reduced in the CT26 + Me49 group (**Figure 1F**). These data indicated that *T. gondii* infection decreased the proportions of G-MDSCs and M-MDSCs in tumor-bearing mice.

In order to investigate whether the Me49 strain of *T. gondii* could directly infect tumor cells *in vivo*, we detected *T. gondii* in different tissues in the CT26 + Me49 group. Results showed that *T. gondii* was detected in the spleen, lung, liver, and brain, but not in tumor tissues (**Supplementary Figure 2**). These results suggested that the increase in CD3^+^CD8^+^ T cells and decrease in CD3^+^CD4^+^ T cells and MDSC *in vivo* might be involved in the antitumoral response of *T. gondii* infection in the mouse model of CRC.

### Characterization of Exosomes From Different Sources

**Figure 2:**
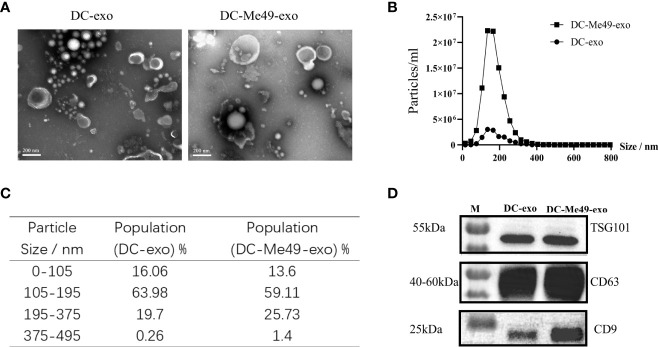
Isolation and characterization of exosomes. **(A)** Exosomes were stained with uranyl acetate and analyzed three times *via* TEM (original magnification, × 100,000; scale bar = 200 nm). **(B)** Nanoparticle-tracking analysis of size distribution of purified exosomes. **(C)** Percentages of purified exosomes in various size ranges. **(D)** Western blot identification of exosome markers CD63, CD9 and TSG101.

Because direct infection with *T. gondii* for cancer therapy can increase the risk of infection, we developed an alternative exosome-based method for CRC treatment. We used ultracentrifugation to obtain exosomes from the culture supernatant of DCs or DCs infected with *T. gondii*. *Via* electron microcopy, we observed the exosomes derived from both kinds of DC to be cup-shaped, with a typical bilayer membrane (**Figure 2A**). DCs infected with Me49 secreted more exosomes at 12 h than uninfected cells (**Figure 2B**). The diameter range of exosomes from different samples was 100–200 nm (**Figure 2C**). The presence of three exosome-enriched proteins, CD63, CD9, and TSG101 (**Figure 2D**), was confirmed by Western blot.

### Exosomes Inhibited Tumor Growth

**Figure 3:**
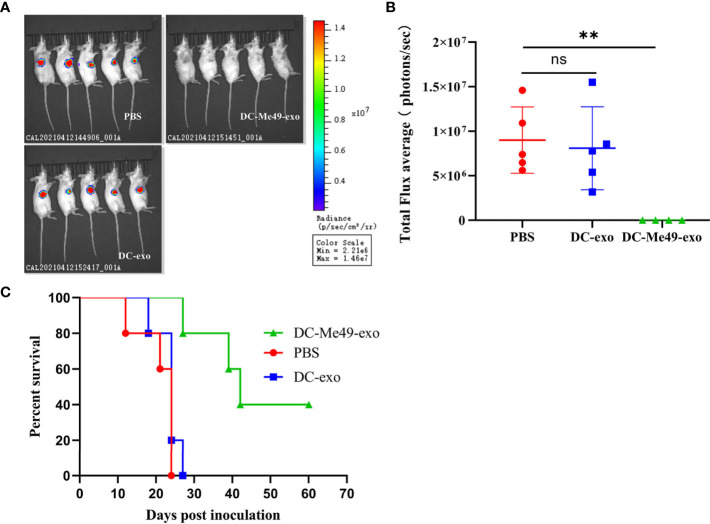
DC-Me49-exo inhibited tumor growth in a mouse model of CRC. CT26 cells were injected subcutaneously, and DC-exo, DC-Me49-exo and PBS from different groups were injected into the tumor. **(A)** Fluorescence intensity in tumor-bearing mice after exosome treatment detected using an IVIS imaging system. **(B)** *In vivo* fluorescence imaging of tumor-bearing mice treated with exosomes. PBS, DC-exo and DC-Me49-exo represent control group, DC-exo treatment group and DC-Me49-exo treatment group, respectively. **(C)** Survival curve of a mouse model with CRC after exosome treatment (n=10). ns, (*p* ≥ 0.05), **p < 0.01.

To evaluate the efficacy of exosomes isolated from DCs infected with *T. gondii*, we treated tumor-bearing mice with exosomes. We monitored tumor growth progression using the IVIS Spectrum. Compared with the DC-exo and PBS groups, signal intensity on tumor imaging was decreased significantly in the DC-Me49-exo group (*P*< 0.01), while no differences were found between the DC-exo and PBS groups (**Figures 3A, B**). DC-Me49-exo group had a 40% survival rate at day 42 versus 0% for the other two groups (**Figure 3C**). These results indicated that DC-Me49-exo could significantly inhibit CRC growth *in vivo*.

### DC-Me49-exo Reduced MDSC Proportion in Tumor Bearing Mice

**Figure 4:**
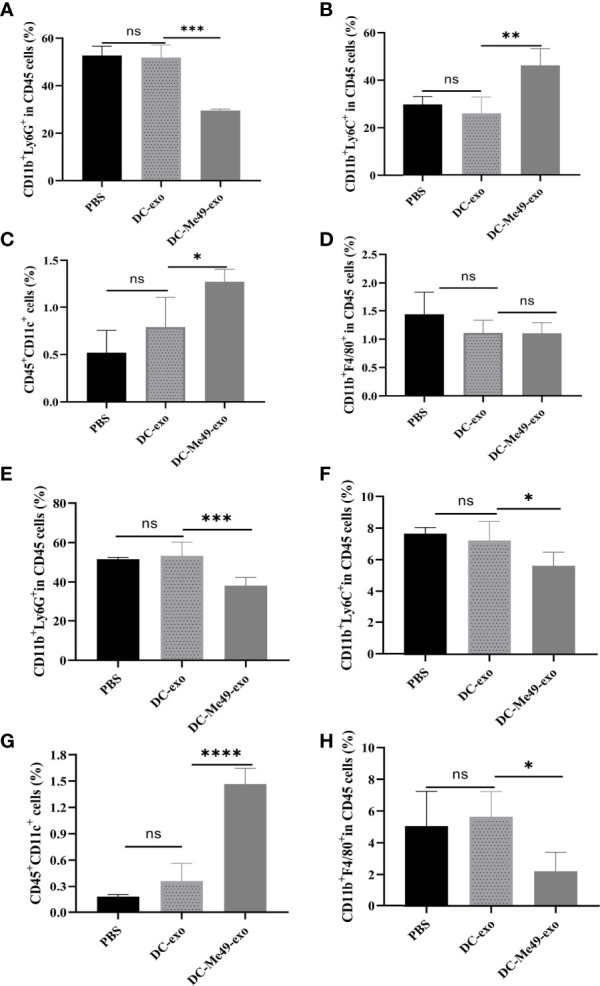
DC-Me49-exo decreased the proportion of MDSCs in a mouse model of CRC. FCM analysis of G-MDSCs (anti-CD11b-APC, anti-Ly6G-PE) **(A)**, M-MDSCs (anti-CD11b-APC, anti-Ly6C-APC-700) **(B)**, DCs (anti-CD45-FITC, anti-CD11c-PC5.5) **(C)** and macrophages (anti-CD11b-APC, anti-F4/80-PB450) **(D)** in the blood of tumor-bearing mice treated with DC-exo, DC-Me49-exo and PBS. FCM analysis of G-MDSCs **(E)**, M-MDSCs **(F)**, DCs **(G)** and macrophages **(H)** in the tumor tissues of tumor-bearing mice treated with DC-exo, DC-Me49-exo and PBS. ns, (*p* ≥ 0.05), *p < 0.05, **p < 0.01, ***p < 0.001, ****p < 0.0001.

To evaluate the role of exosomes in regulating MDSC, we analyzed the level of MDSC in blood and tumors *via* FCM. After CD45^+^CD11b^+^ gating, G-MDSCs, M-MDSCs, DC and macrophage were analyzed (n = 5/group) (**Supplementary Figure 1C**). Results showed that in peripheral blood, the relative proportion of G-MDSCs was reduced after treatment with DC-Me49-exo compared with the DC-exo and PBS groups (*p* < 0.001) (**Figure 4A**). The ratio of M-MDSC was significantly increased (*p* < 0.01) in DC-Me49-exo group (**Figure 4B**). Similarly, compared with the DC-exo and PBS groups, the mean frequency of DCs (CD45^+^CD11C^+^) was significantly increased in the DC-Me49-exo group (*p* < 0.05) (**Figure 4C**). No significant change occurred in the mean frequency of macrophages (CD11b^+^F4/8^+^) in any of the three groups (**Figure 4D**). These data indicated that a significant decrease in G-MDSCs was accompanied by an increase of DCs in the peripheral blood of tumor-bearing mice treated with DC-Me49-exo.

We also analyzed MDSC counts in tumor tissues. We found low proportions of G-MDSCs (*p* < 0.001) (**Figure 4E**) and M-MDSCs (*p* < 0.05) (**Figure 4F**) in the DC-Me49-exo group compared with the DC-exo and PBS groups. Mean frequencies of DCs were significantly increased in the DC-Me49-exo group (*p* < 0.0001) (**Figure 4G**). Compared with the DC-exo and PBS groups, the mean frequency of macrophages was significantly decreased in the DC-Me49-exo groups (*p* < 0.05) (**Figure 4H**). These results indicated that DC-Me49-exo inhibited the accumulation of MDSCs in tumor tissues and peripheral blood, and promoted MDSC (CD11b^+^Ly6G^+^) differentiation.

### DC-Me49-exo Promoted Differentiation of MDSCs *In Vitro*

**Figure 5:**
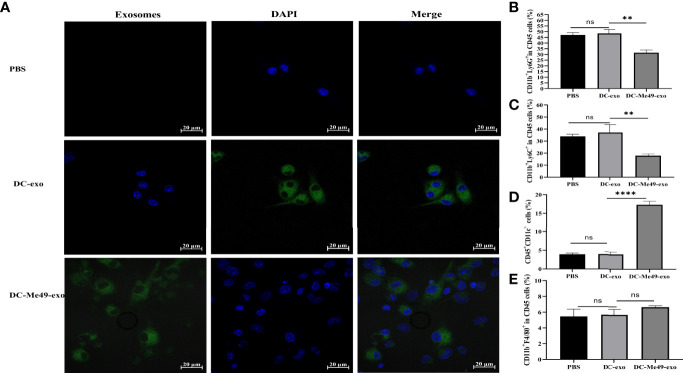
DC-Me49-exo inhibited the proportion of MDSCs *in vitro*. **(A)** Uptake of exosomes by MDSCs. Exosomes were stained green with ExoSparker Exosome Membrane Labeling Kit-Green dye and nuclei were stained blue with DAPI after 24 h co-culture with MDSCs. Scale bar: 20 µm. FCM analysis of G-MDSC (anti-CD11b-APC, anti-Ly6G-PE) **(B)** M-MDSC (anti-CD11b-APC, anti-Ly6C-APC-700) **(C)**, DCs (anti-CD45-FITC, anti-CD11c-PC5.5) **(D)** and macrophages (anti-CD11b-APC, anti-F4/80-PB450) **(E)**. ns, (*p* ≥ 0.05), **p < 0.01, ****p < 0.0001.

Exosomes are taken up by recipient cells and the packaged contents are unloaded to regulate the function and activity of recipient cells. To verify the effect of exosomes on MDSCs, we added labeled DC-exo and DC-Me49-exo to MDSC. After 12 h of coculture, we observed labeled exosomes (green) gathered around the nuclei (blue) of MDSCs (**Figure 5A**), and the proportions of G-MDSCs (*p* < 0. 01) (**Figure 5B**) and M-MDSCs (*p* < 0.01) (**Figure 5C**) were decreased in the DC-Me49-exo group. Compared with the DC-exo and PBS groups, the mean frequency of DC was significantly increased in the DC-Me49-exo group (*p* < 0.0001) (**Figure 5D**). We saw no significant change in macrophage among the three groups (**Figure 5E**). All these data indicated that the DC-Me49-exo directly regulate the MDSC differentiation and increase the level of DCs *in vitro*.

### DC-Me49-Exo Regulated MDSCs by Inhibiting the STAT3 Pathway

**Figure 6:**
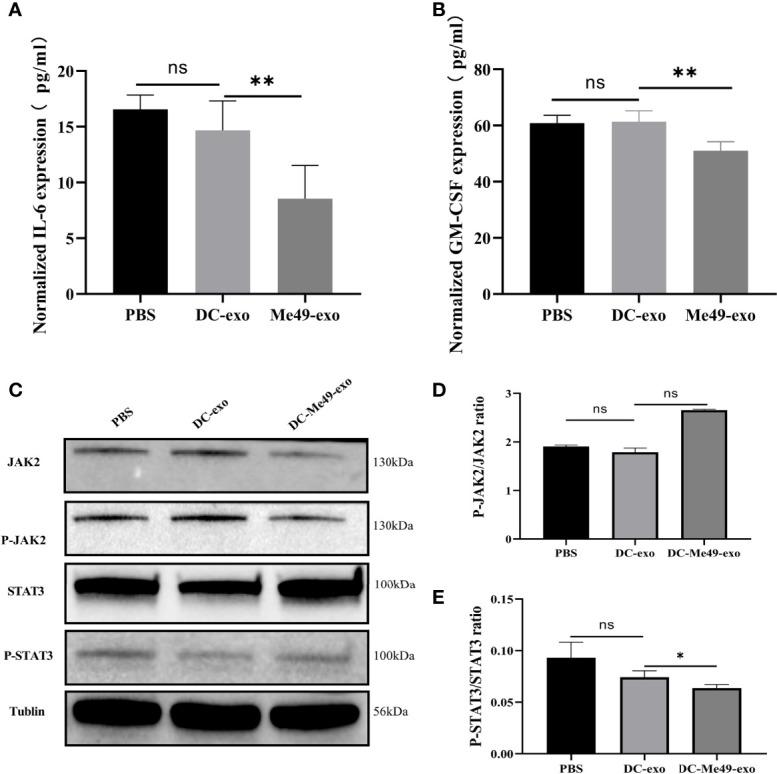
DC-Me49-exo regulated MDSCs by affecting the STAT3 pathway. Concentrations of IL-6 **(A)** and GM-CSF **(B)** in sera of tumor-bearing mice treated with DC-exo, DC-Me49-exo and PBS. **(C)** Protein expression levels at the JAK2/STAT3 pathway. The grayscale analysis of the ratio of P-STAT3/STAT3 **(D)** and P-JAK2/JAK2 **(E)**. ns, (*p* ≥ 0.05), *p < 0.05, **p < 0.01.

Previous studies have confirmed that phosphorylation of JAK2 and STAT3 is associated with the differentiation and expansion of MDSCs (21, 22). We evaluated the levels of IL-6 and GM-CSF, which regulate JAK2–STAT3 activation, in serum of tumor-bearing mice after treatment with different exosomes. Compared with DC-exo and PBS groups, both the levels of IL-6 and GM-CSF significantly decreased in the DC-Me49-exo group (*p* < 0.01) (**Figures 6A, B**). To further confirm the correlation between JAK2-STAT3 signal transduction and MDSC differentiation, we detected phosphorylation of JAK2 and STAT3 in MDSC treated with exosomes using Western blot. The results showed that the phosphorylation level of JAK2 was not affected by exosomes in any of the three groups (**Figures 6C, D**), while that of STAT3 in the DC-Me49-exo group was greatly decreased compared with the DC-exo groups (*p* < 0.05) (**Figures 6C, E**). All these data indicated that DC-Me49-exo promoted MDSC differentiation by inhibiting the phosphorylation level of STAT3.

## Discussion

The oncolytic function of bacteria and viruses has been well studied in cancer therapy. Parasite-based cancer therapy has recently elicited great interest. Previous studies (9, 23) and our own research have found that infection with the single-celled parasite *T. gondii* can inhibit tumor growth. In this study, we detected the distribution of *T. gondii* in tumor-bearing mice, but no parasites were found in tumor tissues. Our hypothesis was that *T. gondii* inhibited tumor growth by rebalancing immune homeostasis. Considering the risk of direct infection with *T. gondii* in tumor treatment, we developed an exosome-based strategy instead of live *T. gondii* infection for cancer therapy. Our data showed that the Me49 strain of *T. gondii* inhibited tumor growth. Compared with the control groups, infiltration of CD3^+^CD8^+^ T cells was increased and the level of MDSC decreased in the CT26 + Me49 group.

Exosomes are secreted by all types of cells including normal, cancer or host, and infected cells, and their functions depend on their cellular origins. Those isolated from different immune cells have been identified, these exosomes have immunomodulatory properties, which encourages research on their clinical applications in disease treatment. At present, the main application of exosomes is tumor prevention. Exosomes derived from immature DCs have limited function, they require activation by antigens or cytokines to exert antitumoral effects (24). Previous studies showed that patients could obtain clinical benefit from exosomes isolated from DCs that were loaded with antigen peptides identified in melanoma and prostate cancer cells (25, 26). Exosomes isolated from DCs co-cultured with human breast adenocarcinoma cells (SK-BR-3) strongly activate tumor-specific T cells (26). These studies show that exosomes derived from DCs are an important new strategy in tumor immunotherapy. Unfortunately, exosomes derived from peptide-loaded DCs have so far failed to induce tumor-specific T cell responses and therefore have no clinical efficacy (7). Compared with exosomes derived from peptide-loaded DCs, exosomes from pathogen-infected DC have a rich variety of antigens, which can bind more effector T cells specifically and produce stronger immune response. *In vivo* experiments, exosomes derived from DC directly loaded with OVA antigen peptide were significantly less able to induce effective antigen-specific T cell response than exosomes derived from DC treated with pulsed OVA. Ova-treated DC exosomes are more sensitive to MHC than exosomes loaded with OVA polypeptides, and have a relatively high affinity for TCR (27). Previous studies (28) and our data proved that *T. gondii* infection could increase CD8^+^ T cell infiltration. DC-derived exosomes display abundant MHC class I/II molecules and T cell co-stimulatory molecules, which mainly perform direct antigen presentation to activate T cells. Therefore, we treated colorectal cancer tumor-bearing mice with *T. gondii* infected DC-derived exosomes. Our results showed that DC-Me49-exo inhibited tumor growth by reducing the level of MDSC in our CT26 mouse model. All these data indicated that exosomes isolated from DCs infected with *T. gondii* could be a potential candidate treatment in cancer therapy. It is a pity that the components of exosomes remain unknown, but we will attempt to clarify them in the future research.

MDSCs, which are major immunosuppressive cells, accumulate in tumor site and promote cancer progression, therefore, targeting them is an attractive strategy for cancer therapy. Our data showed that DC-Me49-exo decreased the level of MDSC both *in vivo* and *in vitro*. A previous study showed that gemcitabine reduced residual G-MDSC in the lung of tumor-bearing mice and inhibited the subsequent metastatic growth (29). In this study, MDSCs and macrophages were significantly reduced in tumor tissues after DC-Me49-exo treatment, and the proportion of DCs increased significantly. These results suggest that this exosome inhibits tumor growth by reducing MDSC at the tumor site. JAK and STAT3 are activated by cytokines and chemokines in the TME, and promote the development of MDSCs (30). *Plasmodium* infection significantly reduces the proportions of MDSCs and regulate T cells (Tregs) in lung tumor tissues of mice by inhibiting phosphorylation of STAT3 and other STAT pathways (12). These findings indicate that inhibiting the proportion and function of MDSCs during tumor progression is essential for tumor treatment. Our data showed that exosomes isolated from *T. gondii*–infected DC significantly inhibited the proportions of MDSCs *in vivo* and *vitro*. However, we focused on MDSC regulation in this study, other immunosuppressive cells such as Tregs, and tumor associated macrophages (TAMs) will be investigated in the future.

In summary, in this study we developed novel pathogen-based exosomes for cancer therapy to replace live-pathogen infection. Exosomes derived from *T. gondii*–infected DCs, therefore, could be a promising therapeutic strategy to inhibit the progression of CRC. The pathogenic infection and the tumor “infection” might competitively regulate the immune system. Exosomes isolated from *T. gondii*–infected DCs, as messengers, could stimulate the immune system and change the “cold” tumor to a “hot” tumor. At the same time, these exosomes could be further modified into carriers for both drug and antibody delivery.

## Data Availability Statement

The original contributions presented in the study are included in the article/**Supplementary Material**. Further inquiries can be directed to the corresponding authors.

## Ethics Statement

The animal study was reviewed and approved by SHVRI–SZ–20200421-01.

## Author Contributions

JL performed experiments, analyzed data and wrote the manuscript. NW conceived the study, performed experiments, analyzed the data. SZ and XC performed experiments. NW, JL, SZ, and XC performed flow cytometry, and conceived and designed the research. HG, RM, and YH suggested the study. GL, NW, and ZC analyzed data, revised manuscript and supervised the study. ZC were responsible for the collection and collation of clinical data. All authors contributed to the article and approved the submitted version.

## Funding

This study was supported in part by the National Natural Science Foundation of China (Grant No. 31302083, 31672541), Shanghai Agriculture Applied Technology Development Program, China (Grant No. 2019-02-08-00-08-F01151), National Key Research and Development Program of China (Grant No. 2017YFD0500401), Shanghai Science and Technology Commission Scientific Research Project (Grant No. 20140900400), and National Risk Assessment Project for Quality and Safety of Agricultural Products (Grant No. GJFP2019027).

## Conflict of Interest

The authors declare that the research was conducted in the absence of any commercial or financial relationships that could be construed as a potential conflict of interest.

## Publisher’s Note

All claims expressed in this article are solely those of the authors and do not necessarily represent those of their affiliated organizations, or those of the publisher, the editors and the reviewers. Any product that may be evaluated in this article, or claim that may be made by its manufacturer, is not guaranteed or endorsed by the publisher.

## References

[1] Pol J Kroemer G Galluzzi L. First Oncolytic Virus Approved for Melanoma Immunotherapy. Oncoimmunology (2016) 5:e1115641. doi: 10.1080/2162402X.2015.1115641 26942095 PMC4760283

[2] Diaz G Wolfe LM Kruh-Garcia NA Dobos KM. Changes in the Membrane-Associated Proteins of Exosomes Released From Human Macrophages After Mycobacterium Tuberculosis Infection. Sci Rep-UK (2016) 6:37975. doi: 10.1038/srep37975 PMC5126699 27897233

[3] Wu ZY Wang LL Li JY Wang LF Wu ZD Sun X. Extracellular Vesicle-Mediated Communication Within Host-Parasite Interactions. Front Immunol (2019) 9:3066. doi: 10.3389/fimmu.2018.03066 30697211 PMC6340962

[4] Tian H Li W. Dendritic Cell-Derived Exosomes for Cancer Immunotherapy: Hope and Challenges. Ann Transl Med (2017) 5 10:221. doi: 10.21037/atm.2017.02.23 28603736 PMC5451628

[5] Bhatnagar S Shinagawa K Castellino FJ Schorey JS. Exosomes Released From Macrophages Infected With Intracellular Pathogens Stimulate a Proinflammatory Response *In Vitro* and *In Vivo* . Blood (2007) 110:3234–44. doi: 10.1182/blood-2007-03-079152 PMC2200902 17666571

[6] Chaput N Thery C. Exosomes: Immune Properties and Potential Clinical Implementations. Semin Immunopathol (2011) 33:419–40. doi: 10.1007/s00281-010-0233-9 21174094

[7] Yao Y Fu C Zhou L Mi QS Jiang A. Dc-Derived Exosomes for Cancer Immunotherapy. Cancers (Basel) (2021) 13(15):3667. doi: 10.3390/cancers13153667 34359569 PMC8345209

[8] Yang Y Liu Q Lu J Adah D Yu S Zhao S. Exosomes From *Plasmodium*-Infected Hosts Inhibit Tumor Angiogenesis in a Murine Lewis Lung Cancer Model. Oncogenesis (2017) 6(6):e351. doi: 10.1038/oncsis.2017.52 28650446 PMC5519199

[9] Parker KH Beury DW Ostrand-Rosenberg S. Myeloid-Derived Suppressor Cells: Critical Cells Driving Immune Suppression in the Tumor Microenvironment. Adv Cancer Res (2015) 128:95–139. doi: 10.1016/bs.acr.2015.04.002 26216631 PMC4662416

[10] Ostrand-Rosenberg S. Myeloid-Derived Suppressor Cells: More Mechanisms for Inhibiting Antitumor Immunity. Cancer Immunol Immunother (2010) 59:1593–600. doi: 10.1007/s00262-010-0855-8 PMC3706261 20414655

[11] Ugel S Delpozzo F Desantis G Papalini F Simonato F Sonda N. Therapeutic Targeting of Myeloid-Derived Suppressor Cells. Curr Opin Pharmacol (2009) 9:470–81. doi: 10.1016/j.coph.2009.06.014 19616475

[12] Adah D Yang YJ Liu Q Gadidasu K Tao Z Yu SL. *Plasmodium* Infection Inhibits the Expansion and Activation of MDSCs and Tregs in the Tumor Microenvironment in a Murine Lewis Lung Cancer Model. Cell Commun Signal (2019) 17(1):32. doi: 10.1186/s12964-019-0342-6 30979375 PMC6461823

[13] Fox BA Sanders KL Chen S Bzik DJ. Targeting Tumors With Nonreplicating *Toxoplasma Gondii* Uracil Auxotroph Vaccines. Trends Parasitol (2013) 29:431–7. doi: 10.1016/j.pt.2013.07.001 PMC3777737 23928100

[14] Riazi Rad F Ajdary S Omranipour R Alimohammadian MH Hassan ZM. Comparative Analysis of CD4+ and CD8+ T Cells in Tumor Tissues, Lymph Nodes and the Peripheral Blood From Patients With Breast Cancer. Iran BioMed J (2015) 19:35–44. doi: 10.6091/ibj.1289.2014 25605488 PMC4322231

[15] Woroniecka K Chongsathidkiet P Elsamadicy A Farber H Cui X Fecci PE. Flow Cytometric Identification of Tumor-Infiltrating Lymphocytes From Glioblastoma. Methods Mol Biol (2018) 1741:221–6. doi: 10.1007/978-1-4939-7659-1_18 PMC6825407 29392704

[16] Roney K. Bone Marrow-Derived Dendritic Cells. Methods Mol Biol (2019) 1960:57–62. doi: 10.1007/978-1-4939-9167-9_4 30798520

[17] Valadi H Ekstrom K Bossios A Sjostrand M Lee JJ Lotvall JO. Exosome-Mediated Transfer of mRNAs and Micrornas Is a Novel Mechanism of Genetic Exchange Between Cells. Nat Cell Biol (2007) 9:654–U72. doi: 10.1038/ncb1596 17486113

[18] Liu D You M Zhao GF Li XJ Song YX Dou H. The Novel Alpha-Glucan Ycp Improves the Survival Rates and Symptoms in Septic Mice by Regulating Myeloid-Derived Suppressor Cells. Acta Pharmacol Sin (2017) 38:1269–81. doi: 10.1038/aps.2017.27 PMC5589966 28649127

[19] Menetrier-Caux C Montmain G Dieu MC Bain C Favrot MC Caux C. Inhibition of the Differentiation of Dendritic Cells From CD34(+) Progenitors by Tumor Cells: Role of Interleukin-6 and Macrophage Colony-Stimulating Factor. Blood (1998) 92:4778–91. doi: 10.1182/blood.V92.12.4778 9845545

[20] Lee CR Kwak Y Yang T Han JH Park SH Ye MB. Myeloid-Derived Suppressor Cells Are Controlled by Regulatory T Cells *via* TGF-Beta During Murine Colitis. Cell Rep (2016) 17:3219–32. doi: 10.1016/j.celrep.2016.11.062 28009291

[21] Nefedova Y Fishman M Sherman S Wang X Beg AA Gabrilovich DI. Mechanism of All-Trans Retinoic Acid Effect on Tumor-Associated Myeloid-Derived Suppressor Cells. Cancer Res (2007) 67:11021–8. doi: 10.1158/0008-5472.Can-07-2593 18006848

[22] Nefedova Y Huang M Kusmartsev S Bhattacharya R Cheng PY Salup R. Hyperactivation of Stat3 Is Involved in Abnormal Differentiation of Dendritic Cells in Cancer. J Immunol (2004) 172:464–74. doi: 10.4049/jimmunol.172.1.464 14688356

[23] Hunter CA Yu D Gee M Ngo CV Sevignani C Goldschmidt M. Cutting Edge: Systemic Inhibition of Angiogenesis Underlies Resistance to Tumors During Acute Toxoplasmosis. J Immunol (2001) 166:5878–81. doi: 10.4049/jimmunol.166.10.5878 11342601

[24] Thery C Regnault A Garin J Wolfers J Zitvogel L Ricciardi-Castagnoli P. Molecular Characterization of Dendritic Cell-Derived Exosomes: Selective Accumulation of the Heat Shock Protein Hsc73. J Cell Biol (1999) 147:599–610. doi: 10.1083/jcb.147.3.599 10545503 PMC2151184

[25] Sedlik C Vigneron J Torrieri-Dramard L Pitoiset F Denizeau J Chesneau C. Different Immunogenicity But Similar Antitumor Efficacy of Two DNA Vaccines Coding for an Antigen Secreted in Different Membrane Vesicle-Associated Forms. J Extracell Vesicles (2014) 3:24646. doi: 10.3402/jev.v3.24646 PMC4149746 25206960

[26] Romagnoli GG Zelante BB Toniolo PA Migliori IK Barbuto JAM. Dendritic Cell-Derived Exosomes May be a Tool for Cancer Immunotherapy by Converting Tumor Cells Into Immunogenic Targets. Front Immunol (2015) 5:692. doi: 10.3389/fimmu.2014.00692 25646096 PMC4298225

[27] Qazi KR Gehrmann U Domange Jordo E Karlsson M Gabrielsson S. Antigen-Loaded Exosomes Alone Induce Th1-Type Memory Through a B-Cell-Dependent Mechanism. Blood (2009) 113(12):2673–83. doi: 10.1182/blood-2008-04-153536 19176319

[28] Tsitsiklis A Bangs DJ Robey EA. CD8(+) T Cell Responses to *Toxoplasma Gondii*: Lessons From a Successful Parasite. Trends Parasitol (2019) 35:887–98. doi: 10.1016/j.pt.2019.08.005 31601477

[29] Bosiljcic M Cederberg RA Hamilton MJ LePard NE Harbourne BT Collier JL. Targeting Myeloid-Derived Suppressor Cells in Combination With Primary Mammary Tumor Resection Reduces Metastatic Growth in the Lungs. Breast Cancer Res (2019) 21:103. doi: 10.1186/s13058-019-1189-x 31488209 PMC6727565

[30] Raychaudhuri B Rayman P Ireland J Ko J Rini B Borden EC. Myeloid-Derived Suppressor Cell Accumulation and Function in Patients With Newly Diagnosed Glioblastoma. Neuro-Onco (2011) 13:591–9. doi: 10.1093neuonc/nor042 PMC3107102 21636707

