# Supplementary material for: Exosomes Derived From Dendritic Cells Infected With Toxoplasma gondii Show Antitumoral Activity in a Mouse Model of Colorectal Cancer
Source: Front Oncol. 2022 May 4;12:899737. doi: 10.3389/fonc.2022.899737 (PMC9114749; doi:10.3389/fonc.2022.899737)

**Supplementary Figure 1.** Gating strategy

**(A)** Flow cytometry analysis of blood, spleen and tumor. Gating strategy for isolation of CD8^+^ T cells and CD4^+^ T cells.

**
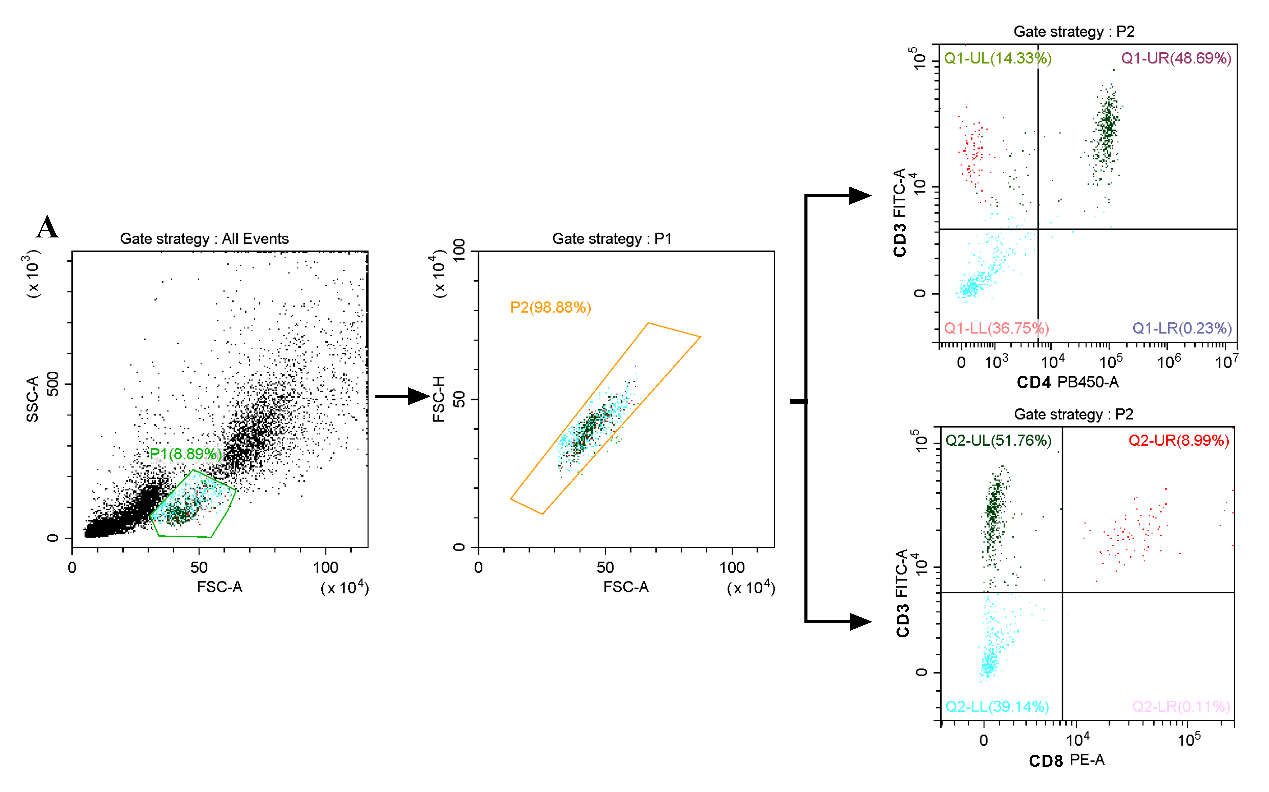
**

**(B)** Flow cytometry analysis of blood, spleen and tumor monocytes gated on CD45^+^CD11b^+^ cells. The experiment was repeated two times. Mean ± SD are shown in the flow plots.


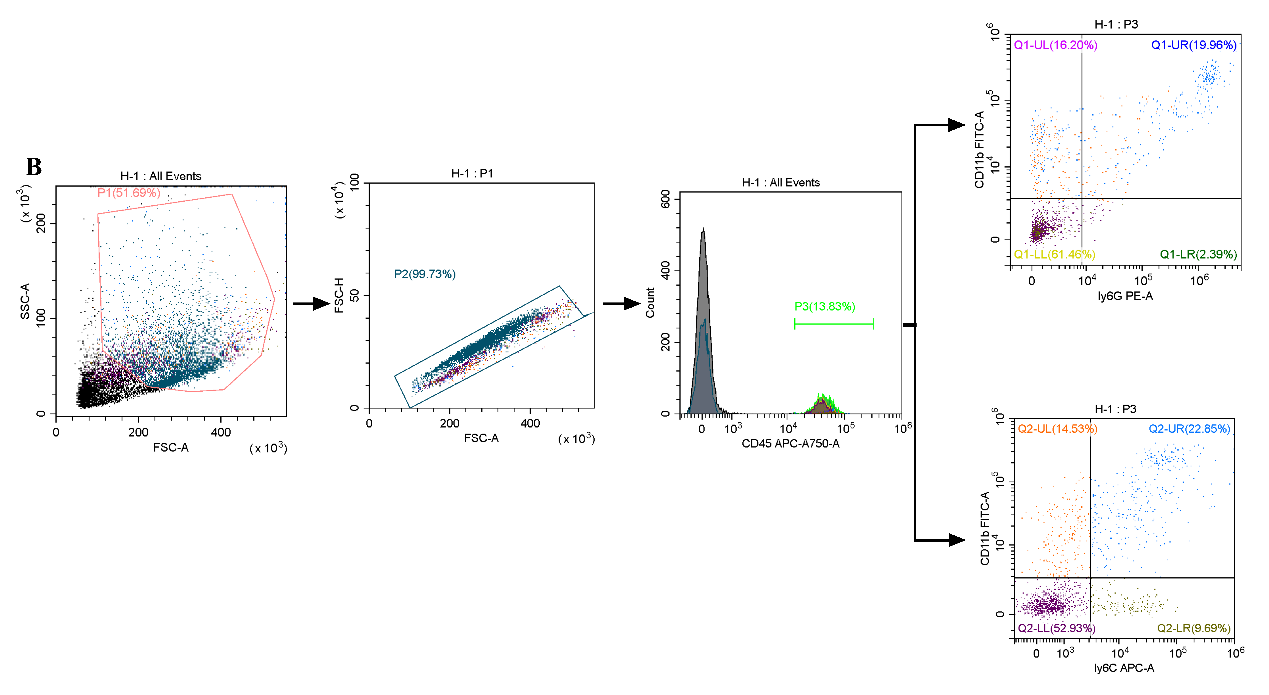


**(C)** Flow cytometry analysis of blood, spleen and tumor monocytes gated on CD45^+^CD11b^+^， CD11b^+^Ly6G^+^, CD11b^+^Ly6C^+^, CD45+CD11c^+^, CD11b^+^F4/80^+^cells (n = 5). The experiment was repeated two times. Mean ± SD are shown in the flow plots.


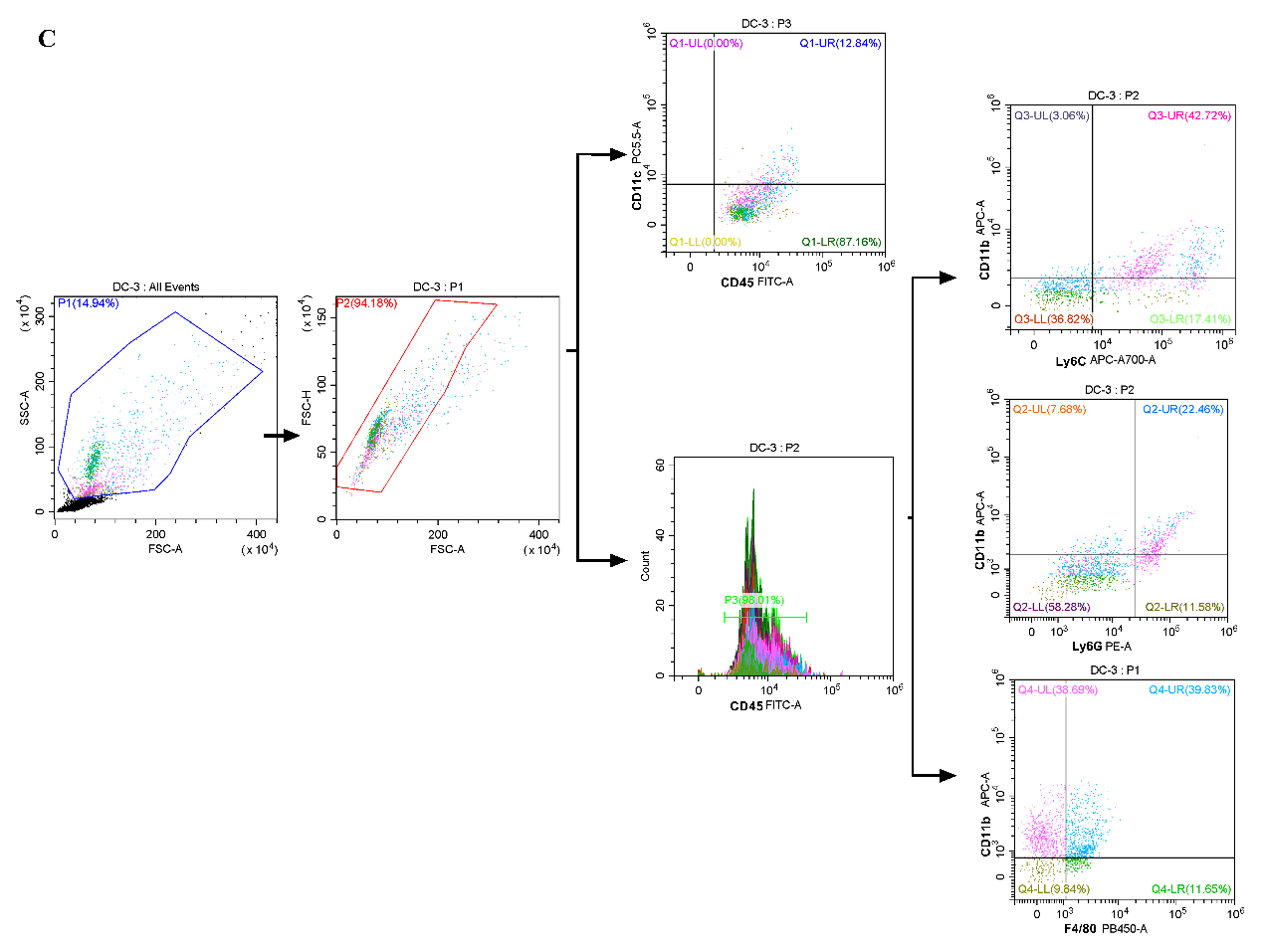


**Supplementary Figure 2.** *T. gondii*-specific DNA copies in different tissues (spleen, lung, liver, brain and tumor). The number of *T. gondii*-specific DNA copies in different tissue was determined by qPCR (n = 4).


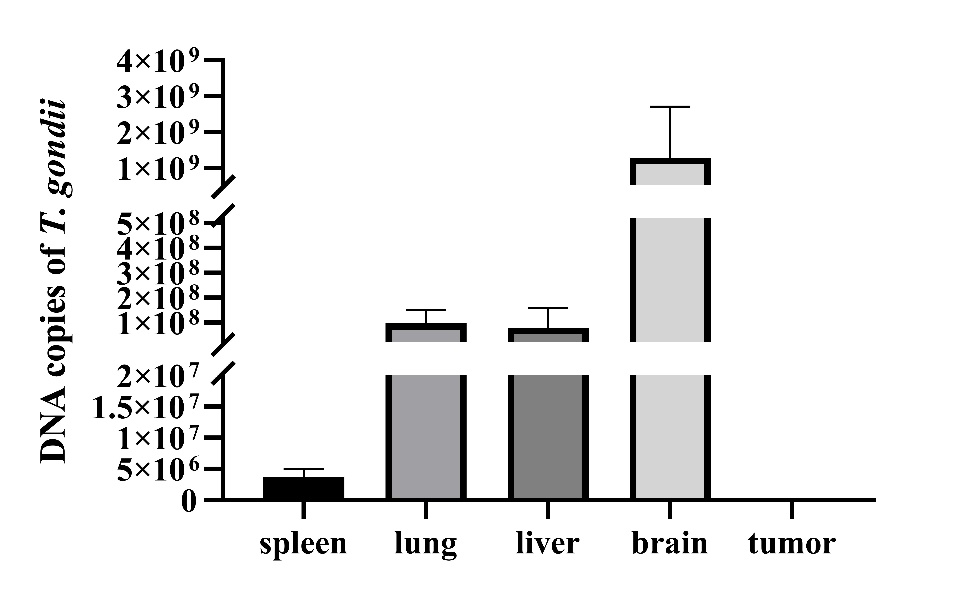

Supplement: Supplementary file 1 [file DataSheet_1.docx]
